# Spontaneous Bone Regeneration in an Open Segmental Fracture of the Forearm with Extruded Middle Segment

**DOI:** 10.7759/cureus.772

**Published:** 2016-09-08

**Authors:** Bibek K Rai, Raju Vaishya, Amit Kumar Agarwal

**Affiliations:** 1 Orthopaedics, Indraprastha Apollo Hospitals

**Keywords:** bone regeneration, distal radius, open segmental fracture, periosteum

## Abstract

Open segmental fractures of both bones of the forearm with the loss of the middle segment of the radius is a rare injury in children. An eight-year-old boy presented to our clinic four days following a road traffic accident. The child’s mother was carrying a 12-cm long extruded and soiled segment of the radius bone. The extruded bone segment seemed necrotic, and we decided not to use it for replantation. The wound over the forearm fracture was infected. It was debrided and regularly dressed until it became healthy. We planned to use a fibular graft for the gap and to fix the graft with a Kirschner wire (K-wire). The operation was delayed due to the poor wound condition. At the four-week follow-up, we noticed unexpected signs of bone regeneration in the bone defect of the radius. After eight weeks, a complete spontaneous reconstruction of the bone was noted. This case highlights the excellent healing potential of the bones in children, where even if a long segment of the bone is lost, we can expect spontaneous complete regeneration of the bone if the periosteum is intact and continuous.

## Introduction

Open segmental fractures of the forearm bones with an extruded long middle segment of the radius is a highly uncommon injury in children. Traumatic bone defects are rare with an estimated frequency of 0.4% for all fractures [1]. Such injuries are more frequent in open fractures than in closed ones. Successful incorporation following the replantation of an extruded bone segment has been reported, but it was reportedly done soon after the injury (i.e. within few hours). In delayed presentations, the surgical options include an induced membrane (i.e. Masquelet) technique, bone transport, bone autograft, vascularized fibular transfer, or no bone reconstruction leaving a single bone forearm [2]. We report a rare case of delayed presentation of extruded middle segment of the radius with successful complete spontaneous bone regeneration in a child, without any intervention. Informed consent was obtained from the patient for this study.

## Case presentation

**Figure 1:**
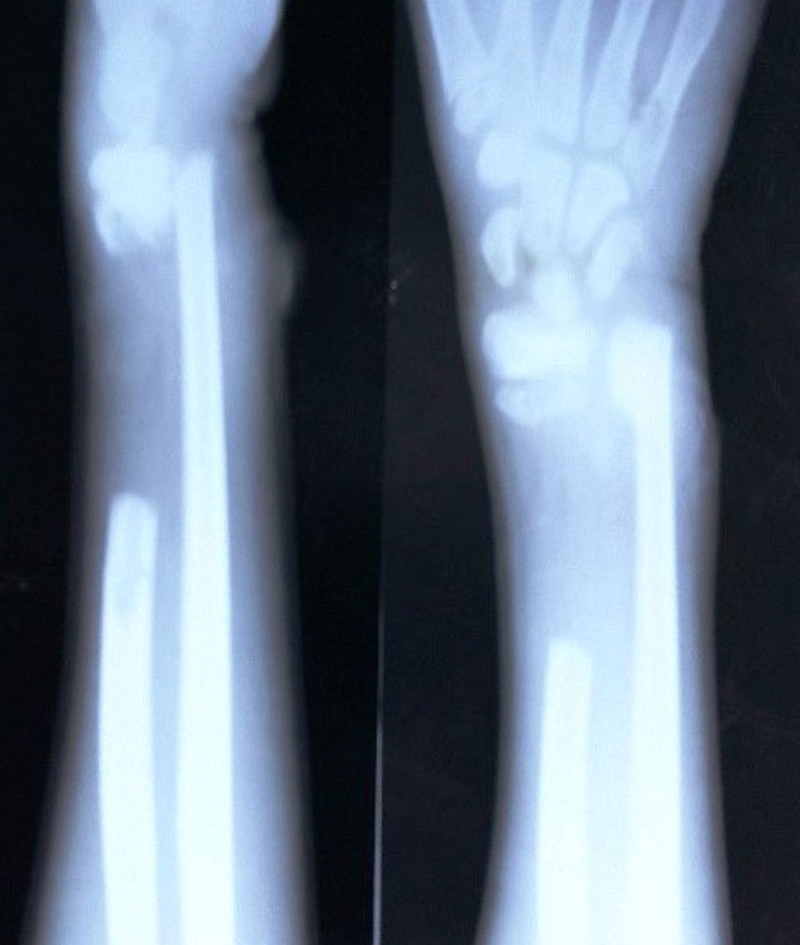
Anteroposterior and lateral X-rays of the forearm with a wrist, at initial presentation four days after injury, showing the 12-cm diaphyseal bone defect of the radius.

An eight-year-old boy presented with pain, swelling, and a lacerated wound on the distal forearm and reported a history of road traffic accident (RTA), four days prior to arriving at our clinic. The local examination revealed swelling, tenderness, deformation, and a 4-cm × 1-cm infected lacerated wound over the volar aspect of the distal forearm. There was no neurovascular deficit. The child’s mother brought a 12-cm extruded and soiled segment of the radius which appeared dry and necrotic. The plain X-rays of the forearm revealed the absence of a middle segment of the radius with a fracture of the distal ulna (Figure 1).

**Figure 2:**
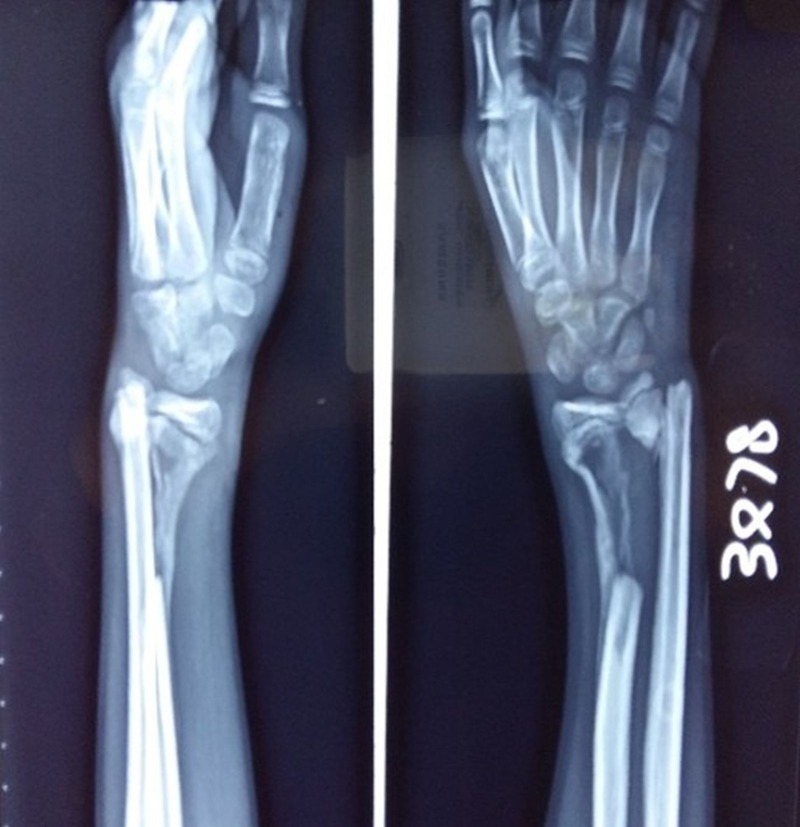
Anteroposterior and lateral radiographs showing the bone formation at four weeks.

The wound was adequately debrided, and the fracture was splinted. We initially planned to do a free fibular graft as the extruded bone was dry and necrotic. The surgery was delayed due to the presence of an infected wound. At the four-week follow-up, a repeat X-ray showed an unexpected callus formation in the diaphyseal bone defect of the radius (Figure 2).

**Figure 3:**
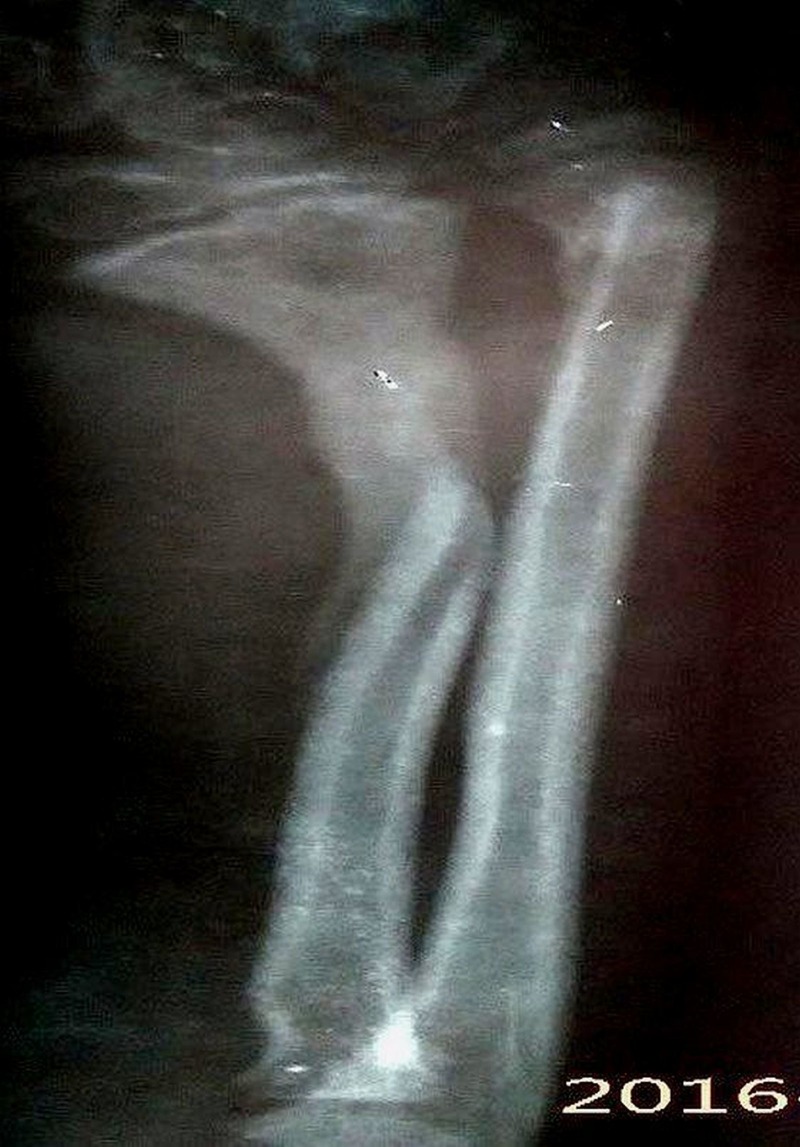
Anteroposterior X-ray of the wrist at eight weeks follow-up showing excellent bone growth in the radial gap.

The patient continued to use an above-elbow brace until the bone had united at eight weeks (Figure 3).

Once the wound healed, regular follow-up evaluations were performed at six, eight, and 12 weeks after the initial injury. At the final follow-up, the range of motion of the forearm, wrist, and elbow were within the functional range without any pain, and the patient had good hand grip strength. A mild radial tilt and positive ulnar variance deformity were present clinically and radiologically, which we planned to treat surgically with corrective osteotomy once the fractures had consolidated.

## Discussion

The segmental loss of a long bone is a challenging clinical problem, as it requires reconstruction of the bone gap. Various treatment methods can be used to manage the bone gap, such as cancellous or corticocancellous autograft, induced membrane technique (i.e. Masquelet) associated with a cancellous autograft, free vascularized bone transfer, and bone transport. Successful incorporation following reimplantation of extruded bone segment soon after the injury has been reported. Kirkup [3] and Abell [4] reported successful replacements of extruded segments of femoral shafts in adults. Tuli, et al. [5] reported a traumatic extrusion of the diaphysis of both the radius and ulna being successfully replaced and maintained by a Plaster of Paris (POP) cast in an eight-year-old child. The process of simultaneous absorption of dead bone and incomplete irregular replacement by new bone has been described in the literature [6].

In our case, we noticed a spontaneous new bone regeneration without any intervention. It might have been possible due to the intact and continuous periosteum in this young child. We did not incorporate the 12-cm long extruded middle segment of the radius as it appeared to be necrotic. In the subsequent months, the X-rays showed an evidence of complete bone growth to fill up the bone defect. A similar open segmental fracture of the radius with an 8-cm defect of the diaphyseal bone has been described in the literature [7]. It was treated by wound debridement and placement of an external fixator (without the interposition of the extruded bone segment). It also showed the complete reconstruction of the bone after two months.

We would like to emphasize that the presence of an intact periosteum is a good prognostic factor in children for bone regrowth [8]. The periosteum is a specialized connective tissue structure covering the bone, and its thickness varies with age. It is thicker, more vascular, active, and loosely attached in infants and children, whereas, in adults it is thinner, inactive, and more firmly adherent. The periosteum is a well-vascularised osteogenic organ responsible for bone growth and thickness via the differentiation of the mesenchymal cells directly into the osteoblasts [9]. The osteogenic potential of the periosteum can become active in a heterotopic environment as long as good vascularization is preserved. The presence of an intact or partially preserved periosteum allows for reconstruction without an additional bone transfer [10]. If a part of the periosteum is intact, an autograft seems sufficient even with extensive bone defects in children. Thus, we believe that due to the presence of the periosteum, the growing bone tissue noted in children has a significant power of regeneration as long as the biological, mechanical, or environmental factors are favorable. Since the presence of infection can have a detrimental effect on bone regeneration despite an intact periosteal sleeve, special care must be taken to treat the infection aggressively. The stability of the fracture is also an essential element for a successful bone reconstruction. In our case, despite the adverse biological and mechanical factors, complete bone growth was achieved.

## Conclusions

In the presence of a thick and intact vascular periosteum, growing bone has significant spontaneous regeneration abilities. This case highlights the excellent healing potential of bones in children, where even if a long segment of the bone is lost, we can expect complete spontaneous regeneration of the bone if the periosteum is intact and continuous.
